# Striatin Is Required for Hearing and Affects Inner Hair Cells and Ribbon Synapses

**DOI:** 10.3389/fcell.2020.00615

**Published:** 2020-07-15

**Authors:** Prathamesh T. Nadar-Ponniah, Shahar Taiber, Michal Caspi, Tal Koffler-Brill, Amiel A. Dror, Ronen Siman-Tov, Moran Rubinstein, Krishnanand Padmanabhan, Chen Luxenburg, Richard A. Lang, Karen B. Avraham, Rina Rosin-Arbesfeld

**Affiliations:** ^1^Department of Human Molecular Genetics and Biochemistry, Sackler Faculty of Medicine and Sagol School of Neuroscience, Tel Aviv University, Tel Aviv, Israel; ^2^Department of Clinical Microbiology and Immunology, Sackler Faculty of Medicine, Tel Aviv University, Tel Aviv, Israel; ^3^Department of Otolaryngology, Head and Neck Surgery, Galilee Medical Center, Nahariya, Israel; ^4^Azrieli Faculty of Medicine, Bar-Ilan University, Safed, Israel; ^5^Goldschleger Eye Research Institute, Sackler Faculty of Medicine, Tel Aviv University, Tel Aviv, Israel; ^6^Department of Cell and Developmental Biology, Sackler Faculty of Medicine, Tel Aviv University, Tel Aviv, Israel; ^7^Visual Systems Group, Abrahamson Pediatric Eye Institute, Division of Pediatric Ophthalmology, Cincinnati Children’s Hospital Medical Center, Cincinnati, OH, United States

**Keywords:** cell junctions, striatin, deafness, hearing loss, STRIPAK

## Abstract

Striatin, a subunit of the serine/threonine phosphatase PP2A, is a core member of the conserved striatin-interacting phosphatase and kinase (STRIPAK) complexes. The protein is expressed in the cell junctions between epithelial cells, which play a role in maintaining cell–cell adhesion. Since the cell junctions are crucial for the function of the mammalian inner ear, we examined the localization and function of striatin in the mouse cochlea. Our results show that in neonatal mice, striatin is specifically expressed in the cell–cell junctions of the inner hair cells, the receptor cells in the mammalian cochlea. Auditory brainstem response measurements of striatin-deficient mice indicated a progressive, high-frequency hearing loss, suggesting that striatin is essential for normal hearing. Moreover, scanning electron micrographs of the organ of Corti revealed a moderate degeneration of the outer hair cells in the middle and basal regions, concordant with the high-frequency hearing loss. Additionally, striatin-deficient mice show aberrant ribbon synapse maturation. Loss of the outer hair cells, combined with the aberrant ribbon synapse distribution, may lead to the observed auditory impairment. Together, these results suggest a novel function for striatin in the mammalian auditory system.

## Introduction

The striatin-interacting phosphatase and kinase (STRIPAK) complex is a multimolecular protein complex involved in numerous biological functions and has been implicated in a number of human diseases (Hwang and Pallas, 2014; Shi et al., 2016; Kuck et al., 2019). STRIPAK complexes regulate phosphorylation of diverse proteins and interact with conserved signaling pathways (Kuck et al., 2019). The mammalian striatin family is ubiquitously expressed and consists of striatin (STRN), SG2NA (STRN3), and Zinedin (STRN4). All these proteins contain multiple WD40 repeats, as well as a Ca^2+^-calmodulin-binding domain, a caveolin-binding motif, and a coiled-coil structure that are essential for their function (Castets et al., 2000; Moreno et al., 2000).

Striatin family members, as part of the STRIPAK complex (Goudreault et al., 2009; Ribeiro et al., 2010; Couzens et al., 2013), are involved in numerous biological processes, ranging from vesicular trafficking (Zhang et al., 2013; Lant et al., 2015) and cell migration (Madsen et al., 2015; Bazzi et al., 2017) to regulating the hippo signal transduction pathway (Chen et al., 2019; Tang et al., 2019). Striatin is expressed in diverse cellular and subcellular compartments such as the medium spiny neurons in the striatum (Benoist et al., 2006) and membrane-bound signaling complexes (Lu et al., 2004). Other studies, as well as ours, have shown that striatin is also expressed in junctions between epithelia cells (Breitman et al., 2008; Domke et al., 2014; Franke et al., 2015; Domke and Franke, 2019; Lahav-Ariel et al., 2019). Interestingly, striatin has been shown to be expressed in different types of cell junctions. In three different human cancer-originating epithelial cell lines, Caco2 and CFAPAC-1, striatin co-localizes with the TJ marker ZO-1 but not with the AJ protein-β-catenin (Breitman et al., 2008). In another cancer-originating cell line, striatin was found to localize with TJ proteins but not with the AJ protein E-cadherin (Lahav-Ariel et al., 2019). In human colon epithelial sections, mouse testis tissues and lamellar smooth muscle cells striatin colocalizes with the plaque protein P0071 (Domke et al., 2014; Franke et al., 2015; Domke and Franke, 2019). Striatins colocalize with E-cadherin and with plaque proteins, such as β-catenin and P0071, as well as in lateral AJs and in plaques of some forms of tessellate junctions (Franke et al., 2015).

The cochlea, part of the mammalian inner ear involved in hearing, contains distinct inner hair cells (IHCs) and outer hair cells (OHCs) which convert mechanical stimuli into electrical signals (Dallos, 1992). Cell–cell junctions in the organ of Corti within the cochlea are highly complex and crucial for the ability of mammals to hear. In the cochlea, adherens junctions (AJs) are associated with inner ear physiological processes, while tight junctions (TJs) are important for maintaining ion concentration gradient between the endo- and perilymph (Wang et al., 2015). The mammalian inner ear sensory organ epithelial cells consist mainly of hair cells and supporting cells. TJs and AJs interconnect hair cells and supporting cells, generating the reticular lamina and have been referred to as hybrid tight-adherens junctions (TAJs), as they are not distinct (Nunes et al., 2006). These junctions are critical for providing a barrier between the perilymph and endolymph, which differ in K+ and Na+ concentrations. Endolymph, high in K+ but low in Na+, contacts the apical surfaces of hair cells and supporting cells, while perilymph, low in K+ and high in Na+, bathes the basolateral surfaces of these cells (Wan et al., 2013). This difference generates an electrochemical gradient across the reticular lamina (dependent on the TAJs), resulting in the endocochlear potential (EP) necessary for hair cell depolarization and normal hearing. The integration of striatin into the AJ zonula plaques before formation of the mature junction at a stage where they are closely associated with TJ proteins (Franke et al., 2015) suggest that striatin may be a component of different cell–cell junctions, a feature that may depend on cell and tissue time or developmental stage.

Here, we examined the localization and function of striatin in the mouse cochlea in order to evaluate its role in the auditory system. Our results show that striatin is specifically expressed in cell junctions between the IHCs of the organ of Corti, and that striatin knockdown leads to progressive hearing loss. Although loss of striatin did not lead to changes in the junctional integrity of the hair cells, or affect the EP, partial degradation of the OHCs was observed, particularly in the basal region of the organ of Corti. This observation is consistent with our results showing that striatin-deficient mice exhibit high-frequency hearing loss. Normal hearing requires proper positioning of the ribbon synapses that undergo complex organizational processes during the maturation of IHCs within the organ of Corti (Liberman, 1982; Liberman et al., 2011; Yin et al., 2014; Kalluri and Monges-Hernandez, 2017). Our results show that striatin-deficient mice have a uniform distribution of ribbons that lack the spatial gradient seen in wild-type IHC. Interestingly, a similar aberrant maturation of the synapse is seen in striatin binding protein adenomatous polyposis coli (APC) mutant mice (Hickman et al., 2015). Taken together, our results present striatin as a novel multifunctional protein that is essential for mammalian hearing.

## Materials and Methods

### Strn-Knockout Mice: Establishment and Genotyping

The ES cell line EPD0082_3_E07 carrying the Strn^*tm1a(KOMP)Wtsi*^ allele was injected into embryos, which were transplanted into recipient C57BL/6 female mice. All animal procedures were approved by the Animal Care and Use Committee (IACUC) at Tel Aviv University (01-18-085) and Cincinnati Children’s Hospital Medical Center (3D09062). Genotyping was performed from tail samples by PCR, using a set of primers that flank the *Strn* gene: F-5′TTCCTTTGAGAAAACACAGTCCCAG-3′, R′-5′-ACACACTCCACTGAACAAAGTCAAGC-3′, to give a 1257bp product in the wild-type mice and a set of primers that flank the LoxP-common forward primer 5′-GAGATGGCGCAACGCAATTAAT-3′ and gene specific reverse primer 5′-ACACACTCCACTGAACAAAGTCAAGC-3′, to give a product of 437 bp in homozygous mutants, with both products present in heterozygous littermates.

### Auditory Brainstem Response

To investigate auditory function and phenotype, ABR tests were performed on P20, P30, P40, and P60 mice using tone-burst stimuli. Briefly, mice were anesthetized by intraperitoneal injection of xylazine (20 mg/ml at 5% v/v) and ketamine (100 mg/ml at 10% v/v) administered at the rate of 0.1 ml per 10 g body mass, and placed in an acoustic chamber (MAC-1, Industrial Acoustic Company), as previously described (Horn et al., 2013).

### Scanning Electron Microscopy

Mice inner ears were dissected in cold PBS buffer shortly after mice were euthanized by CO_2_ inhalation. The temporal bone was removed prior to overnight fixation in glutaraldehyde (2.5% v/v in PBS) at 4°C. The samples were alternately incubated in osmium tetroxide and thiocarbohydrazide after exposing the organ of Corti, as previously described (Hunter-Duvar, 1978). After treatment, the samples were vacuum dried and mounted on a metal plate. Subsequently the samples were gold-coated at the Faculty of Life Sciences Electron Microscopy Unit at Tel Aviv University and imaged with a JSM 540A scanning electron microscope (Jeol).

### Western Blot Analysis

Cochlea and Huh7 cell protein lysates were prepared using Nonidet P-40 lysis buffer [150 mM NaCl, 1.0% Nonidet P-40, Tris–Cl (50 mM pH 8.0) protease inhibitor mixture, for 30 min on ice. The lysate was cleared by centrifugation at 13200 rpm for 15 min at 4°C, and supernatant was recovered. Protein concentration was determined using the BCA protein determination reagent (Sigma), and 50 μg were resolved on an SDS/PAGE denaturing gel and transferred to a nitrocellulose membrane. Immunoblots were performed using the appropriate antibodies, and the membranes were developed using the Quantum ECL detection kit (K-12042-D20; Advansta). The immunoblot bands were quantified using ImageJ software, and the variation in protein loading was corrected by normalization to the levels of the indicated loading control protein such as tubulin. For IP, the primary antibody was incubated with protein A/G agarose beads (Santa Cruz Biotechnology, Dallas, TX, United States) at 4°C with mild shaking. 2 mg of cleared lysate was precleared with protein A/G agarose beads for 1 h at 4°C and incubated overnight with antibody-conjugated protein A/G agarose beads at 4°C. Beads were recovered and washed five times with lysis buffer before resolving in SDS-PAGE. Subsequently IP was confirmed with the appropriate antibody.

### Cochlea Protein Extraction

Total protein from cochlea was extracted as previously described (Bhonker et al., 2016). Briefly, 12 cochleas from wild-type P0 mice were dissected and lysed with 10% NP-40 protease inhibitor mixture, kept for 30 min on ice, and centrifuged at 13200 rpm for 15 min at 4°C, to harvest the supernatant. Protein concentration was determined using the BCA protein determination reagent (Sigma), and 60 μg were resolved on an SDS/PAGE gel and transferred to a nitrocellulose membrane. Immunoblots were performed using the appropriate antibodies. The membranes were developed using the WesternBright Quantam kit (K-12042-D20; Advansta, San Jose, CA, United States).

### Tail Protein Extraction

To confirm the genotyping, total protein was homogenized from the tails using BioVortexer (BioSpec Products, Bartlesville, OK, United States) and 120 μg of protein was resolved on an SDS/PAGE, as subjected to Western blot analysis.

### Immunolocalization

Whole mount immunohistochemistry of inner ear was performed as previously described (Dror et al., 2010). Briefly, the inner ears were dissected in cold PBS buffer shortly after P30 mice were euthanized by CO_2_ inhalation. Temporal bone was removed prior to overnight fixation in paraformaldehyde (4% v/v in PBS) at 4°C. The sensory epithelium was fine dissected, blocked, and permeabilized by incubation in blocking buffer (normal goat serum in 0.1% triton) for 2 h at room temperature. Samples were incubated with indicated primary antibody overnight at 4°C. After a brief wash in PBSX1, samples were incubated with secondary antibody for 2 h at room temperature. To visualize F actin, this was followed by incubation for 1 h at room temperature in phalloidin conjugated to Alexa Fluorophores (Life Technologies). The stained samples were mounted on Histobond microscope slides (Marienfeld GmbH) using Prolong Gold (Thermo Scientific) and dried overnight at room temperature. Image acquisition was performed with a confocal laser microscopy system (LSM800 Carl Zeiss).

### Endocochlear Potential Recording

Mice were anesthetized by intraperitoneal injection of 50 mg/kg pentobarbital. The skin covering the neck was cut to expose the trachea. A tracheostomy was performed in order to maintain sufficient ventilation. The cochlea was exposed by a ventral approach and the tympanic bulla was gently picked to expose the cochlea. A drill was then used to expose the spiral ligament beneath the lateral wall. A glass pipette filled with 150 mM KCl was gradually inserted into the scala media through the spiral ligament while continuously recording the DC potential. The EP was defined as the delta between the potential recorded in the scala media and the one recorded on the spiral ligament. Potentials were amplified by OC-725C (Warner Instruments, CT, United States), digitized at 1 kHz using MiniDigi 1A (Molecular Devices, CA, United States) and analyzed using pCLAMP 9 (Molecular Devices).

### Antibodies

The following antibodies were used for this study: rabbit anti-striatin (IB: 1:1,000; IHC 1:250; Proteintech) mouse anti-striatin (IB: 1:1000; IHC: 1;250; BD Transduction Laboratories), mouse anti-Ctbp2 (IHC 1:250; BD Transduction Laboratories), rabbit anti-myosin VIIa (IHC 1:250; Proteus Biosciences), mouse anti-ZO1 (IHC: 1:100; Thermo Scientific), mouse anti-PP2A (IB:1:1000; Upstate Biotechnology), rabbit anti-striatin 4 (IHC: 1:250; Abcam), rat anti-Ecad (IHC:1:250; Santa Cruz), Phallodin-488 (IHC: 1:1000; Abcam), mouse anti-Alexa fluor 594 (IHC: 1:250; Abcam), rabbit anti-Alexa fluor 633 (IHC: 1:250; Invitrogen); mouse anti-tubulin (IB 1:10,000; Sigma) was used as a loading control.

### Quantification of Ribbon Synapses

Whole mount immunohistochemistry was performed as described. The region between the first and second turn of the cochlea was dissected and stained with Ctbp2 and myosin VIIA antibodies. The samples were carefully mounted to avoid excessive pressure from the coverslip that can squeeze the tissue. Image acquisition was performed with a confocal laser microscopy system (LSM800, Carl Zeiss, Oberkochen, Germany). The Z stacks of the images were exported to Imaris software (Zurich, Switzerland). All the analyses were performed using the same settings in Imaris. For P17, 4–11 IHCs, and for P35, 5–10 IHCs, from each cochlea were quantified after exporting the file to Imaris.

### Barrier Function Assay

The TJ tracer permeability assay was performed as previously described (Furuse et al., 2002). Briefly, 50 μl of 10 mg/ml EZ-Link Sulfo-NHS-LC-Biotin (Thermo Scientific) in 1 mM CaCl_2_/PBS was injected into the dermis on the back of the *Strn*^+/+^ and *Strn*^–/–^ newborns. After 30-min incubation, the skin was removed and frozen in liquid nitrogen. Frozen sections were fixed for 30 min at 4°C in 95% ethanol, followed by 1 min incubation at room temperature in 100% acetone. The sections were then incubated for 15 min in blocking solution followed by labeling with anti-oclludin (Thermo Scientific; 1:100) for 30 min. The sections were subsequently washed and incubated for 30 min with the secondary antibody (Invitrogen; 1:250) and streptavidin Texas red (Thermos Scientific). Image acquisition was performed using a confocal laser microscopy system.

## Results

### Striatin Is Expressed in the Apical Surface of the Inner Hair Cells

**FIGURE 1:**
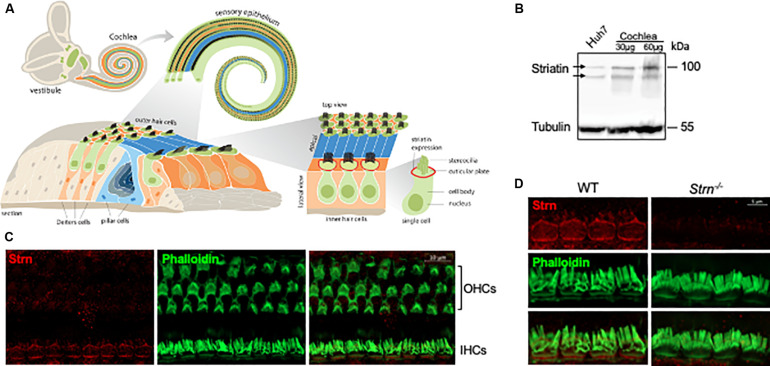
Striatin is expressed in cell–cell junctions of the mouse inner hair cells. **(A)** Schematic representation of the mouse inner ear. **(B)** Striatin is expressed in the cochlea. Cochlea and Huh7 (as a positive control) cell lysates were analyzed by SDS-PAGE and immunoblotted using the indicated antibodies. Tubulin served as a loading control. **(C)** Striatin is expressed in the inner hair cells (IHC). Immunofluorescence analysis of striatin expression in P30 whole-mount mouse inner ears stained with striatin, and phalloidin to visualize filamentous actin. **(D)** Striatin is localized to the sites of cell–cell junctions in IHCs. Immunofluorescence analysis performed on WT and *Strn^–/–^* littermates show that striatin is localized to the sites of cell–cell junctions between the IHCs.

Depending on cell type and condition, mammalian striatin localizes to diverse subcellular compartments such as the Golgi (Frost et al., 2012), endoplasmic reticulum, plasma membrane, mitochondria (Tanti and Goswami, 2014; Jain et al., 2017), and cellular junctions (Lahav-Ariel et al., 2019). As cell–cell adhesion is essential for auditory function, and since striatin had low diffuse expression in the cochlear sensory epithelium in the early postnatal stages, with a higher expression detected in inner and outer hair cells in the maturing cochlea (Rudnicki et al., 2014; Chessum et al., 2018; Ranum et al., 2019)^1^, we were interested in examining the expression pattern and role of striatin in the auditory system. To this end, we designed and constructed a striatin-deficient mouse (Supplementary Figure S1). Cochlea of P0 mice were dissected and total protein was extracted for Western blot analysis. The specific striatin knockdown was verified by Western blot analysis of wild-type (WT), *Strn^+/–^* and *Strn^–/–^* littermates (Supplementary Figure S2A). As expected, there was no immunostaining of striatin in the *Strn^–/–^* mice. We further examined the expression and localization of striatin in the inner ear; a schematic illustration of the cochlea, including the localization of striatin, is shown in Figure 1A. Results shows that striatin is indeed expressed in the cochlea at the protein level (Figure 1B). The localization of striatin in the organ of Corti was evaluated by immunofluorescence assays. Here, striatin was specifically detected in the cell–cell junctions of the IHCs (Figures 1C,D).

### Striatin Knockout Mice Exhibit Progressive Hearing Loss

**FIGURE 2:**
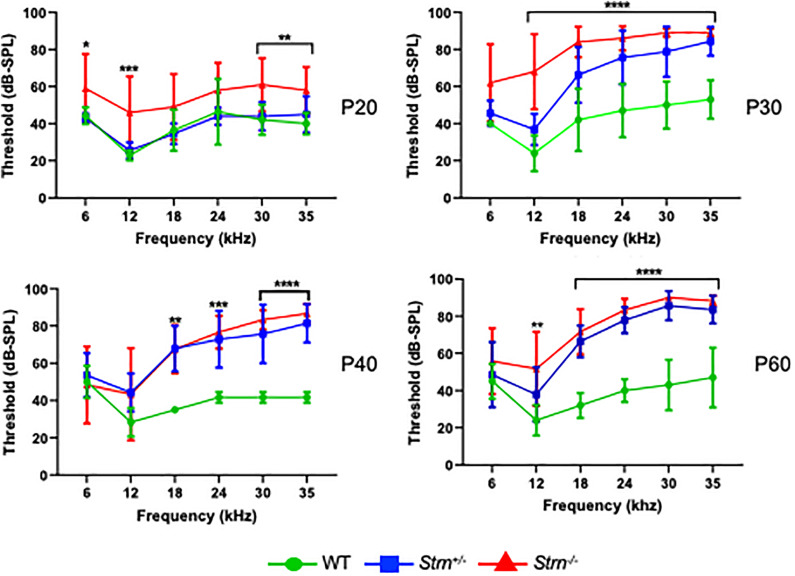
Auditory brainstem response (ABR) reveals progressive hearing loss in *Strn^–/–^* mice at higher frequencies. ABR threshold means are shown for *Strn^–/–^* mice and littermate control mice, tested at the indicated ages. P20: *n* = 10 *Strn^+/–^*, 5 *Strn^–/–^*, and 7 WT, P30: *n* = 8 *Strn^+/–^*, 5 *Strn^–/–^*, and 5 WT, P40: *n* = 7 *Strn^+/–^*, 6 *Strn^–/–^*, and 3 WT, and P60: *n* = 7 *Strn^+/–^*, 6 *Strn^–/–^*, and 5 WT. Data shown as mean ± SEM. Statistical tests were two-way ANOVA with Holm-Sidak multiple comparison correction. *P < 0.1, **P < 0.01, ***P < 0.001, ****P < 0.0001.

To further understand the function of striatin in the auditory system, we used the Auditory Brainstem Response (ABR) recordings. ABR was performed on P20, P30, P40, and P60 WT, *Strn^+/–^*, and *Strn*^–/^*^–^* littermates using a sound stimulus with varying frequencies (6–35 kHz) and intensities (10–90 dB). The results showed that the hearing threshold for *Strn^+/–^* mice was similar to that of the WT mice at P20, and that there was a modest shift in hearing thresholds for certain frequencies in the *Strn*^–/^*^–^* mice compared to the WT mice. However, at P30, there was a substantial increase in the hearing thresholds of both the homozygous and heterozygous striatin mice, compared to the WT mice. At P40 and P60, both *Strn^+/–^* and *Strn^–/–^* mice showed severe hearing loss, indicating that striatin deficiency leads to progressive hearing impairment (Figure 2). Representative ABR traces in response to 30 kHz sound stimuli is presented in Supplementary Figure S3. The striatin mutant mice had compromised hearing at frequencies above 12 kHz, implying that the mid and basal regions of the organ of Corti could be affected.

### Striatin Mutants Show Moderate Outer Hair Cell Degeneration

**FIGURE 3:**
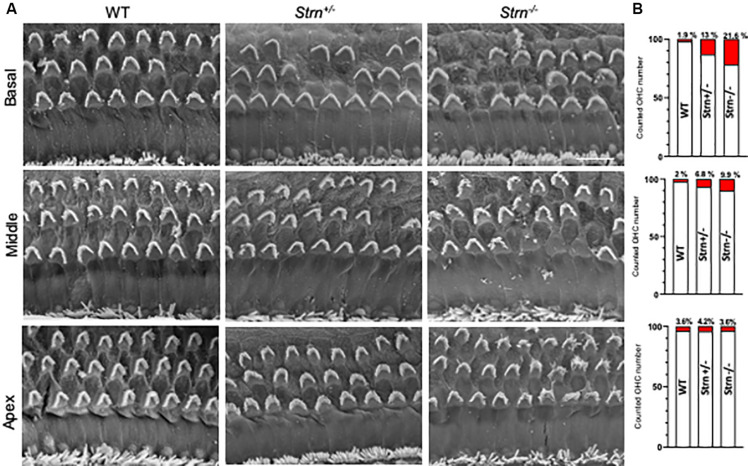
Scanning electron micrograph and quantification of hair cells show moderate outer hair cell (OHC) degeneration in *Strn^–/–^* mice. The hair cells were counted in 200 μm stretches of middle and basal coil regions at P60. **(A)** Representative electron micrograph reveals moderate hair cell degeneration of OHC in *Strn^+/–^* and *Strn^–/–^* as compared to WT in the middle and basal region of the organ of Corti. **(B)** The graphs show numbers of cell loss/survival for middle and basal regions. Middle coil region (upper panel): 2/87 (Control, *n* = 3), 7/103 (*Strn^+/–^*, *n* = 4), 28/284 (*Strn^–/–^, n* = 12), and basal coil region (lower panel): 4/206 (Control, *n* = 8), 43/274 (*Strn^+/–^*, *n* = 11), 32/148 (*Strn^–/–^*, *n* = 7). Cell loss is marked in red.

To better understand the observed hearing loss, we examined hair cell degradation using scanning-electron microscopy (SEM). The results show moderate OHC degeneration in *Strn^+/–^* and *Strn^–/–^* mice as compared to WT mice (Figure 3A). Quantification of the hair cells revealed 21.6%, 9.9%, and 3.6% loss of OHCs in the basal, mid and apical regions of the cochlea, respectively, for *Strn^–/–^* mice, and 13%, 6.8% and 4.2% loss of OHCs in the basal, mid and apical regions, respectively, for *Strn^+/–^* mice (Figure 3B).

### Cell–Cell Junctional Integrity Is Mostly Retained in the Striatin Knockout Mice

**FIGURE 4:**
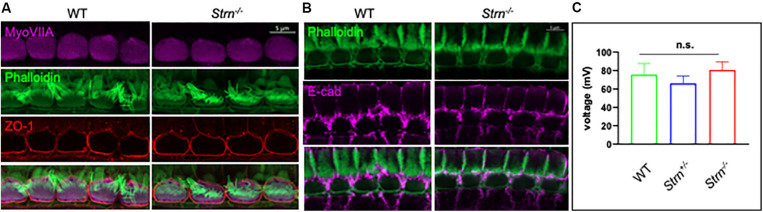
Junctional integrity in the organ of Corti is intact in *Strn^–/–^* mutants. **(A,B)** Representative P30 whole-mount mouse inner ear stained with myosin VIIa (purple), ZO1 antibody (red) and phalloidin (green) **(A)**, or E cadherin (purple) and phalloidin (green) **(B)**. The experiments were repeated using 4–5 mice of each genotype. **(C)** Recordings of endocochlear potential (EP) of endolymph are shown for *Strn^–/–^* and littermate control mice, tested in 3-month old mice (*Strn^–/–^* (*n* = 2), *Strn^+/–^* (*n* = 7) and WT (*n* = 3). Statistical test was one-way ANOVA with Holm-Sidak correction for multiple comparisons.

Cell–cell junctions in the cochlea are crucial for maintaining the correct structure and function of the organ of Corti and complete or partial loss of function of cell junction proteins often results in impaired hearing (Kitajiri et al., 2014; Higashi et al., 2015; Kamitani et al., 2015; Kazmierczak et al., 2015). Striatin was shown to maintain junctional integrity in cultured mammalian cells (Lahav-Ariel et al., 2019) which accords with our finding of striatin in IHC cell junctions (Figure 1). Immunostaining was used to monitor the expression pattern and subcellular localization of the tight junctional (TJ) protein ZO-1 and the adherence junction protein E-cadherin in the hair cell junctions of striatin knockout mice. The expression pattern of both ZO-1 and E-cadherin in the knockout mice was similar to that of WT mice, indicating that the junctional integrity in the organ of Corti is maintained in striatin-deficient mice (Figures 4A,B).

The stria vascularis produces the endolymph extracellular fluid (Patuzzi, 2011) and generates an EP that is essential for normal auditory function (Quraishi and Raphael, 2008; Xiong et al., 2011). The TJs in the organ of Corti are required to form the cation-junctional barrier between perilymph and endolymph and to maintain the EP (Florian et al., 2003; Kitajiri et al., 2004). The presence of striatin at the junctions of the IHCs led us to examine whether striatin knockout mice are capable of maintaining the EP. No significant changes were detected in the EP of striatin-deficient mice (Figure 4C). This finding is in accordance with our results showing that the junctional integrity between neighboring IHCs is not compromised by striatin loss.

To examine the effect of striatin loss on TJs, a barrier function assay in P1 mice was performed (Furuse et al., 2002). We injected an isotonic solution containing a primary amine-reactive biotinylation reagent, Sulfo-NHS-LC-Biotin, subcutaneously, into the back of *Strn*^+/+^ and *Strn*^–/–^ newborns, and following incubation the skin was dissected out and frozen. Frozen sections were double labeled with anti-occludin and streptavidin to detect TJs and bound biotin, respectively (Supplementary Figure S4). In the wild-type mice epidermis, the tracer’s diffusion was stopped by the TJs (defined by the occludin staining), whereas in the striatin null mice, occludin was somewhat mislocalized (white arrow) and we observed a slight diffusion of the Sulfo-NHS-LC-Biotin tracer through the occludin expression sites (rectangle; arrow in rectangle indicates accumulation of occludin). This result suggests that the permeability of the TJs was slightly disrupted due to striatin loss.

### Striatin Knockout Mice Display an Aberrant Ribbon Gradient

Striatin is a subunit of the serine/threonine phosphatase PP2A (Moreno et al., 2000), and reduced PP2A activity impairs synaptic function (Viquez et al., 2009). Interestingly, lack of another member of the STRIPAK complex, striatin interacting protein 2 (Strip2), leads to a decrease in neural response amplitudes. Since the synaptic ribbons of IHCs express Ctbp2 (Khimich et al., 2005; Pisciottano et al., 2019), we examined the expression pattern of Ctbp2 in P17 and P35 mice, time points at which striatin-deficient mice exhibit hearing loss. To examine the synaptic ribbons of IHCs, the cochlear region at the junction between the first and second cochlear turns, which corresponds to ∼16 kHz hearing frequency, where the ribbon size gradients are most pronounced (Liberman et al., 2011), were selected for quantitative image analysis (Hickman et al., 2015).

**FIGURE 5:**
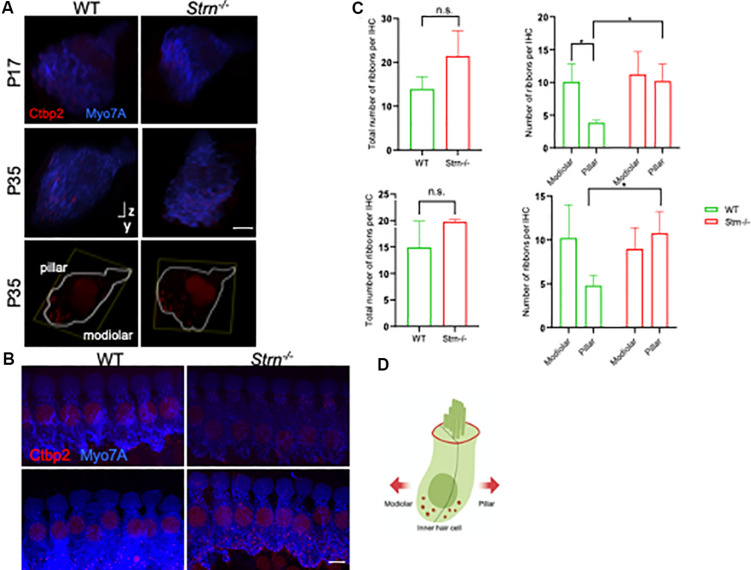
*Strn^–/–^* mice lack a correct ribbon size localization gradient. Confocal micrographs of ribbons at the synaptic poles of individual IHCs (Ctbp2, red, and myosin VIIa, blue) in z-stacks, oriented on the *y*–*z*-axis. **(A)** Representative side view of at least eight contiguous IHC in WT and *Strn^–/–^* P17 and P35 mice. Bottom panels: representative side view of at least two IHCs stained at P35 with Ctbp2 (outlined in white based on myosin VIIa staining). **(B)** Representative maximum projection of myosin VIIa (blue) and Ctbp2 (red) in IHC, with the latter staining synaptic poles of IHCs for WT and *Strn^–/–^* P17 mice. **(C)** Total ribbon number and ribbon gradient along the modiolar and pillar regions in *Strn^–/–^* mice and WT littermates. Imaris software was used to analyze the 3D z-stacks, 4-11 IHCs from each cochlea were imaged for quantitative ribbon analysis. Statistical analysis was performed by 2-way ANOVA and Holm-Sidak multiple comparisons test (*n* = 4). P17, upper panel; P35 lower panel. **(D)** Schematic diagram of an IHC demonstrating plane of orientation of pillar vs. modiolar region.

Z-stacks of at least eight continuous IHCs were imaged in WT and striatin null mice. Myosin VIIa was used to stain the hair cell body. The 3D structures were analyzed using Imaris software, followed by quantification of the number and localization of the Ctbp2 puncta with respect to the modiolar and pillar faces of the IHC. Figure 5 shows a representative 3D side view of the IHCs that were used to determine the puncta number. Although the total number of Ctbp2 puncta at the synaptic poles of the *Strn^–/–^* mice at two time points, P17 and P35, was not significantly different from that of WT mice (Figure 5D), the Ctbp2 puncta in *Strn^–/–^* mice showed a uniform distribution and lack the ribbon localization gradient toward the modiolar side of the IHC, as seen in WT mice that show a spatial gradient toward the modiolar face of IHC (Figures 5A,C).

## Discussion

The STRIPAK complex, which includes striatin, a subunit of the PP2A enzyme, is associated with numerous biological roles ranging from cell signaling to developmental processes (Kuck et al., 2019). To study the biological function of striatin, a knockout mouse was generated. The *Strn^–/–^* mice are viable; however, they suffer from progressive hearing loss, especially at high frequencies. At P20, the mice have only a mild hearing loss; however, at P30, the hearing threshold increases substantially. By P60, the mice exhibit severe hearing loss above 12kHz. Although OHC loss in the *Strn^–/–^* mice was not substantial relative to the wild-type mice, this loss might contribute to the higher frequency hearing loss of the mutant mice. However, these results imply that hair cell degradation is not the only cause of the severe hearing loss we observed. OHC loss could also be secondary to initial damage to other components of the auditory system.

In the mammalian cochlea, the IHCs and OHCs are the two anatomically and functionally distinct types of mechanosensitive receptor cells (Dallos, 1992) and each cell type expresses distinct genes (Liu et al., 2014; Li et al., 2018; Ranum et al., 2019). Examining the expression pattern of striatin in the inner ear revealed that that in the cochlea, striatin is specifically expressed in the cell junctions between IHCs and is not expressed in the OHCs. Genes specifically associated with IHCs were shown to function in neurotransmitter vesicle transport and fusion (Liu et al., 2014) and interestingly, phocein, a neuronal striatin-interacting protein, has been shown to be important for synaptic plasticity and is involved in vesicular trafficking (Bailly and Castets, 2007).

Cell–cell junctions in the cochlea are highly complex and are crucial for maintaining the correct structural and functional integrity of the cochlea (Wang et al., 2015). As striatin is important for the integrity of these junctions (Lahav-Ariel et al., 2019), we examined the expression pattern of ZO-1 and E-cadherin that mark tight and adherence junctions, respectively. Our results demonstrate that lack of striatin did not affect the localization of these junctional proteins and the normal EP in the *Strn^–/–^* mice indicates that junctional integrity was not compromised. One possible explanation may be the presence of a redundant protein compensating for striatin loss. Striatin 4 shares over 50% protein sequence homology with striatin and also binds the catalytic subunit of PP2A, indicating that the proteins may have redundant cellular functions, as seen in various systems (Hwang and Pallas, 2014; Kazmierczak-Baranska et al., 2015).

In addition, striatin has been shown to colocalize with β-catenin, a junctional protein, in different tissues (Domke et al., 2014; Franke et al., 2015; Domke and Franke, 2019). However, as our previous studies indicate that in the mammalian cells tested striatin did not bind or colocalize with β-catenin (Breitman et al., 2008) and that in the inner ear β-catenin is barely seen in hair cells (Liu et al., 2016), the possibility that striatin and β-catenin interact was not examined in the current study. Another interesting prospect is the effect of striatin on proto-cadherins. Proto-cadherins are a diverse group of adhesion molecules that belong to the cadherin family, which are mainly expressed and function in the nervous system (Morishita and Yagi, 2007). In the human inner ear, protocadherin-15 (PCDH15) and cadherin-23 (CDH23) form a filament called the tip-link that connects adjacent stereocilia of mechanosensitive hair cells that is essential for hearing (Pickles et al., 1984; Kachar et al., 2000; Jaiganesh et al., 2018b). The flexibility and elasticity of PCDH15 is crucial for the function of these tip-links (Powers et al., 2017; Bartsch et al., 2019). Over 100 mutations of the tip link cadherins are associated with hearing loss in humans (Jaiganesh et al., 2018a), in which most affect elastic properties of the link and reduce Ca^2+^ affinity and unfolding strength of the protein (Sotomayor et al., 2010). However, as PCDH15, which is expressed in the neurosensory epithelium of the ear, does not appear to be expressed in the junctions between hair cells (Ahmed et al., 2003) and as mice that express a mutated PCDH15 protein display abnormal hair bundles in both inner and outer hair cells, a phenotype not observed in the *Strn* mutant (Alagramam et al., 2011), we speculate that the effect of striatin depletion on the auditory system is not mediated through proto-cadherins.

Following the observation that in *Strn^–/–^* mice there is hearing loss that cannot be explained by OHC loss, and the fact that striatin localizes to IHC, we wanted to understand whether striatin deficiency impairs IHC function. We and others have shown that striatin interacts with APC (Breitman et al., 2008; Tran et al., 2008). As APC is expressed in the inner ear epithelial cells (Mogensen et al., 2002), as well as in efferent olivocochlear neurons, and the finding that APC affects synaptic maturation, leading to reduced dynamic ranges of hearing probably affecting the correct IHC ribbon size gradient (Hickman et al., 2015), we examined IHC ribbon synapses. Interestingly, it has recently been shown that knockdown of Strip2 in the inner ear leads to a reduction in the number of afferent synapses, suggesting a potential cochlear neuropathy (Pisciottano et al., 2019).

An analysis of Ctbp2 puncta in *Strn^–/–^* mice showed a lack of the ribbon localization gradient toward the modiolar side of the IHC, relative to the wild-type mice This finding suggests aberrant maturation of the ribbon structure in striatin-deficient mice, which subsequently could affect auditory function. Interestingly, in APC, another striatin-interacting protein, deficient mice (Breitman et al., 2008; Tran et al., 2008), ribbon synapses were shown to lack a size gradient toward the modiolar face of the IHCs (Hickman et al., 2015). APC conditional knock-out mice show impaired auditory function, which probably results from aberrant afferent synapse ribbon size gradients. Similarly to the *Strn^–/–^* mice, the IHCs of the APC knock-out mice show wild-type ribbon numbers but lack the normal ribbon size gradient (Hickman et al., 2015). As ribbon synapses mediate transmission between IHCs and spiral ganglion neurons (SGNs), our results lead us to propose that striatin, a component of the STRIPAK complex that interacts with the tumor supressor protein APC, regulates auditory synapses. This finding is supported by the observation that the *Drosophila* striatin homolog, CKA, facilitates axonal transport of dense core vesicles and autophagosomes in a PP2A-dependent manner (Neisch et al., 2017). Moreover, as striatin functions as a PP2A subunit, it is also plausible to speculate that lack of striatin will disrupt the phosphorylation patterns that are essential for precise auditory function (Niceta et al., 2015; Xie et al., 2017; Zhai et al., 2018). The specific role of striatin in IHC ribbon synapse development is not clear, and since striatin appears to be localized to the junctions of IHC and not the ribbons themselves, its function in this respect is likely indirect.

In this context, another STRIPAK component, Strip2 (Goudreault et al., 2009; Kean et al., 2011), which is expressed in OHC and IHC (Scheffer et al., 2015; Pisciottano et al., 2019; Ranum et al., 2019), is known to be important for neural response amplitudes (Pisciottano et al., 2019).

OHCs induce cochlear amplification in the mammalian inner ear (Ashmore, 1987; Dallos, 1992) and it has been shown that prestin is required for the electromotility of the OHC, which is believed to be a major component of the cochlear amplifier (Liberman et al., 2002; Dallos, 2008). Different lines of evidence suggest that OHCs are more vulnerable than IHCs to noise and age (Liberman and Kiang, 1978; Wu et al., 2019), and loss of OHCs leads to elevated sound detection thresholds. It is important to note that in mice lacking TJs, there is an increased concentration of K^+^ around the basolateral surfaces of the OHCs, resulting in hair cell degeneration (Ben-Yosef et al., 2003; Nakano et al., 2009; Nayak et al., 2013; Morozko et al., 2015; Kitajiri and Katsuno, 2016). Moreover, USP53, a TJ protein in cochlear epithelial cells, affects the survival of OHCs (Kazmierczak et al., 2015). However, since the OHC loss in striatin null mice is very mild and the hearing loss is very severe, and more importantly since striatin is not detected in OHC, we believe that OHC loss is merely a secondary phenomenon to a primary defect in other cells. As knockdown of APC that interacts with striatin (Breitman et al., 2008; Tran et al., 2008) from olivocochlear (OC) neurons leads to lack of the normal ribbon-size gradient in IHCs and reduction of the dynamic range of hearing (Hickman et al., 2015), we suggest that the observed change in distribution of ribbons between the modiolar and pillar side of IHCs may be the primary cause of hearing loss in the striatin null mice.

Furthermore, a recent study shows that disruption of cochlear gap junction-mediated intercellular communication (GJIC) in the spiral ligament specifically induces OHC cell death (Nishiyama et al., 2019). The damaging effects of aminoglycoside antibiotics on the cochlea are also much more extensive on OHCs as compared to their effects on IHCs (Xiong et al., 2011). Interestingly, in mice lacking the Kcc4 co-transporter that is expressed in hair and supporting cells, at P21, OHCs were almost totally lost, whereas IHCs were still present (Boettger et al., 2002).

In conclusion, our results provide the first evidence that striatin, a member of the conserved STRIPAK complex, functions in the auditory system. Although we hypothesize that the role of striatin may be related to maturation of IHC ribbon synapses and cell–cell junctions, other roles are also possible, making this finding an exciting opportunity for further investigation and validation.

## Data Availability Statement

The raw data supporting the conclusions of this article will be made available by the authors, without undue reservation.

## Ethics Statement

The animal studies were reviewed and approved by Tel Aviv University and the Cincinnati Children’s Hospital Medical Center.

## Author Contributions

KA and RR-A conceived the project. RL generated the Striatin knockout mice. PN-P designed and performed the genotyping, dissections of inner ears, ABR, SEM, confocal imaging, Western blots, and quantification of ribbon synapses. MR and ST performed the endocochlear potential experiments. CL and KP conducted the biotin barrier assay. ST and TK-B aided with dissections and SEM imaging analysis. MC and AD aided with image analysis, experimental design and organizing the data. RS-T helped with the mice work and analysis. PN-P, ST, KA, and RR-A wrote the manuscript. KA and RR-A supervised the work. All authors contributed to the article and approved the submitted version.

## Conflict of Interest

The authors declare that the research was conducted in the absence of any commercial or financial relationships that could be construed as a potential conflict of interest.

## Footnotes

1 umgear.org

## Supplementary Material

FIGURE S1 Generation of the striatin knockout mice line. **(A)** Schematic representation of the striatin gene. Rectangles represent exons, with coding sequence colored in orange. **(B)** The first and second exons are shown. Green arrowheads are FRT sites. Blue arrowheads denote LoxP sites. Recombination of the FRT sites flanking the Neo results in the floxed Strn allele. Recombination of the LoxP sites removes the ATG start site in exon 1 resulting in the *Strn* null allele P1, P2, and P3 represent the location of the primers used for *Strn* genotyping. **(C)** For genotyping, tails were excised from mice and genomic DNA was extracted and subjected to PCR analysis. Primers were designed to generate amplicons of 1.2 kb for the WT and 450 bp for the null mutant.

FIGURE S2 The expression of striatin 4 is unaffected in *Strn^–/–^* mice. Total protein extracted from tails was resolved and immunoblotted for Western blot analysis, using the indicated antibodies. **(A)** Validation of mice genotyping using an anti-striatin antibody that detects only the Strn1 isoform. **(B)** Western blot analysis shows that the expression of striatin 4 is unaffected in *Strn^–/–^* mutants at P58. Tubulin was used as loading control.

FIGURE S3 Representative ABR waves at 30 kHz showing increased threshold for *Strn^–/–^* as compared to the control.

FIGURE S4 Biotin tracer TJ permeability assay. Freshly prepared isotonic solution of biotin was injected into the dermis of P1 *Strn*^+/+^ and *Strn*^–/–^ mice. Frozen sections were double stained with anti-occludin and streptavidin Texas red to label TJs and the cross-linked biotin tracer. The white arrow indicates mislocalization of occludin in the mutant mice; the rectangle marks an epidermis section in which leakiness of occludin can be observed in the mutant.
