# Supplementary figures and images for: Striatin Is Required for Hearing and Affects Inner Hair Cells and Ribbon Synapses
Source: Front Cell Dev Biol. 2020 Jul 15;8:615. doi: 10.3389/fcell.2020.00615 (PMC7381154; doi:10.3389/fcell.2020.00615)

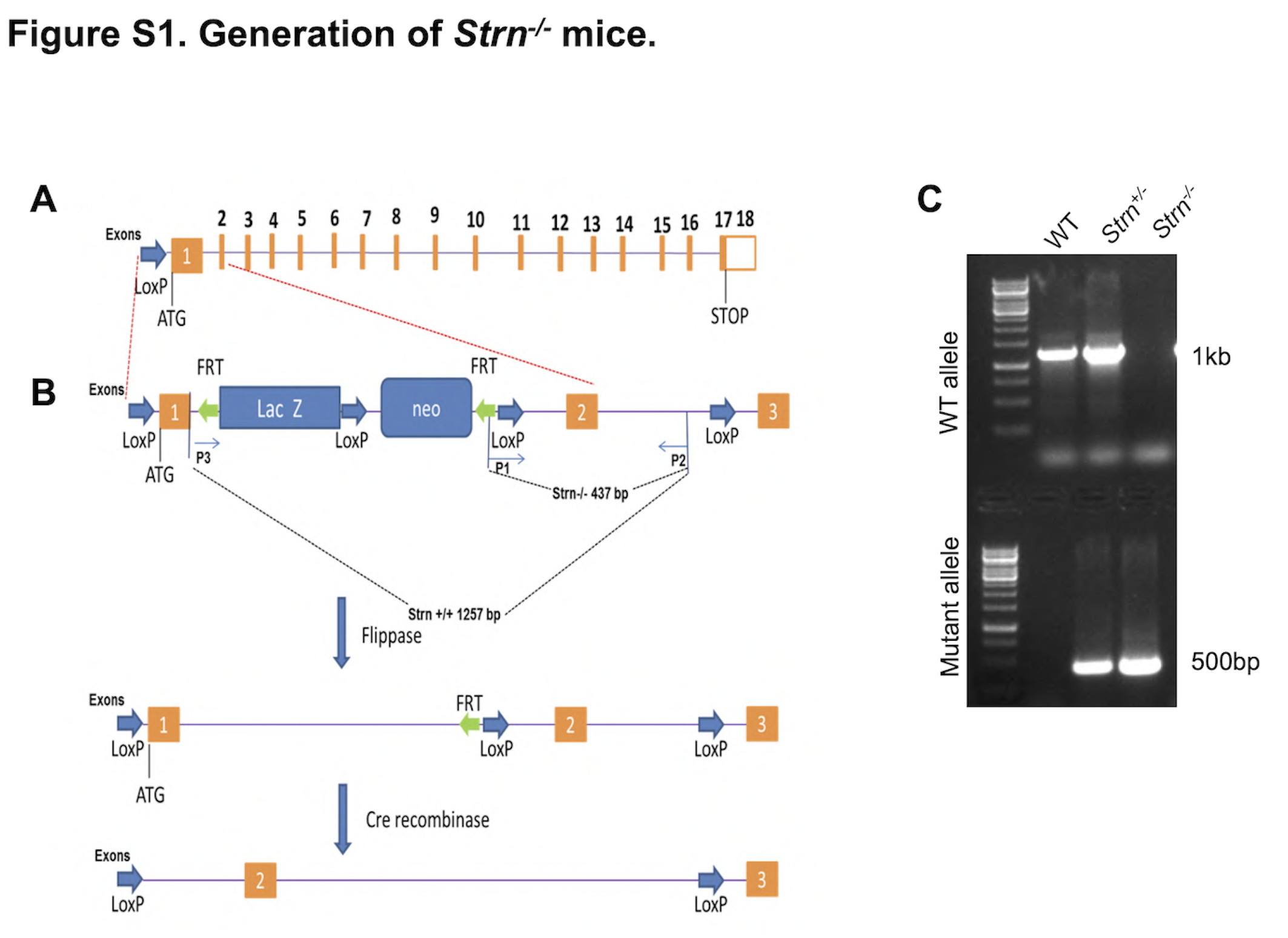

Supplement: FIGURE S1 — Generation of the striatin knockout mice line. (A) Schematic representation of the striatin gene. Rectangles represent exons, with coding sequence colored in orange. (B) The first and second exons are shown. Green arrowheads are FRT sites. Blue arrowheads denote LoxP sites. Recombination of the FRT sites flanking the Neo results in the floxed Strn allele. Recombination of the LoxP sites removes the ATG start site in exon 1 resulting in the Strn null allele P1, P2, and P3 represent the location of the primers used for Strn genotyping. (C) For genotyping, tails were excised from mice and genomic DNA was extracted and subjected to PCR analysis. Primers were designed to generate amplicons of 1.2 kb for the WT and 450 bp for the null mutant. [file Image_1.TIFF]

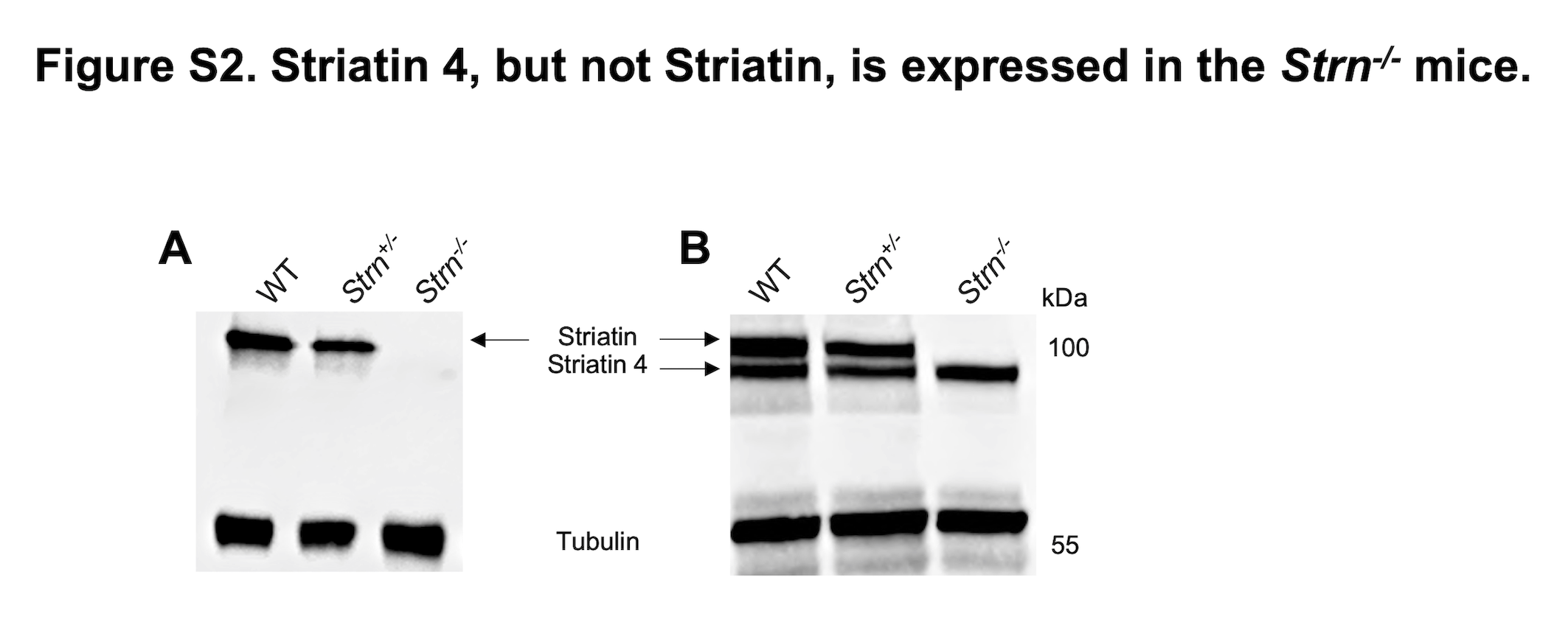

Supplement: FIGURE S2 — The expression of striatin 4 is unaffected in Strn–/– mice. Total protein extracted from tails was resolved and immunoblotted for Western blot analysis, using the indicated antibodies. (A) Validation of mice genotyping using an anti-striatin antibody that detects only the Strn1 isoform. (B) Western blot analysis shows that the expression of striatin 4 is unaffected in Strn–/– mutants at P58. Tubulin was used as loading control. [file Image_2.TIFF]

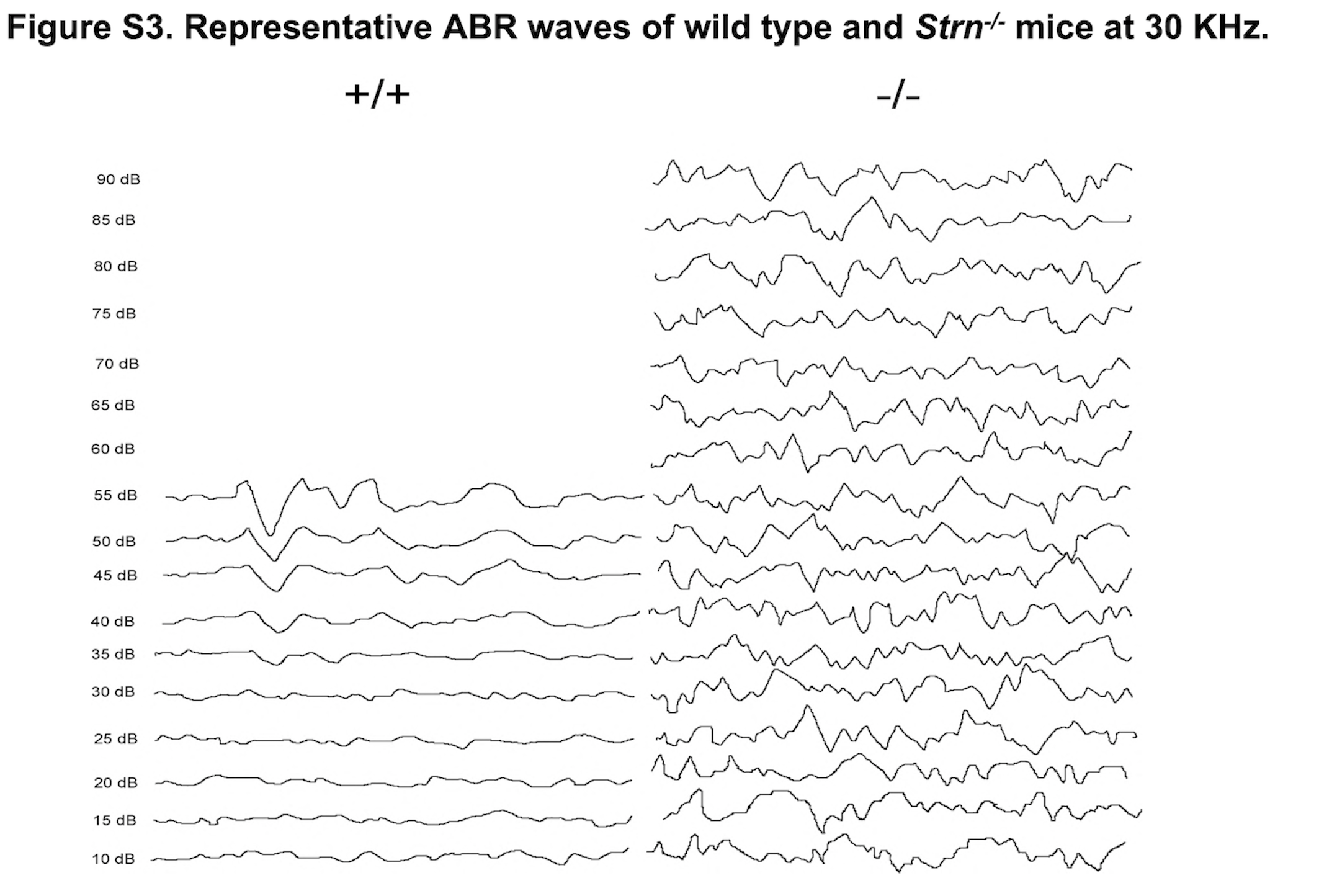

Supplement: FIGURE S3 — Representative ABR waves at 30 kHz showing increased threshold for Strn–/– as compared to the control. [file Image_3.TIFF]

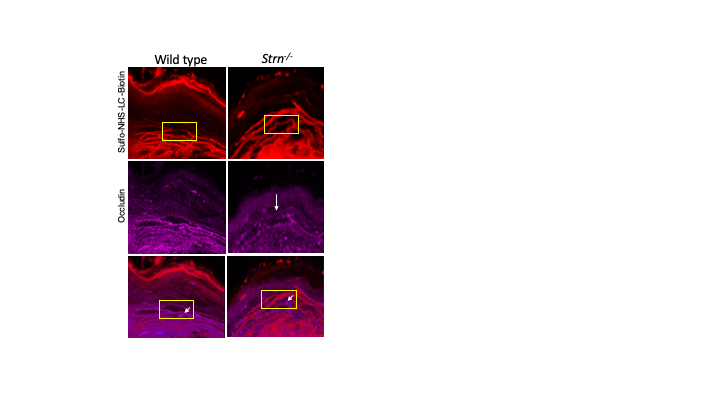

Supplement: FIGURE S4 — Biotin tracer TJ permeability assay. Freshly prepared isotonic solution of biotin was injected into the dermis of P1 Strn+/+ and Strn–/– mice. Frozen sections were double stained with anti-occludin and streptavidin Texas red to label TJs and the cross-linked biotin tracer. The white arrow indicates mislocalization of occludin in the mutant mice; the rectangle marks an epidermis section in which leakiness of occludin can be observed in the mutant. [file Image_4.TIFF]
